# The evolution of menopause in cetaceans and humans: the role of demography

**DOI:** 10.1098/rspb.2010.0988

**Published:** 2010-06-30

**Authors:** Rufus A. Johnstone, Michael A. Cant

**Affiliations:** 1 Department of Zoology, University of Cambridge, Cambridge CB2 3EJ, UK; 2 Centre for Ecology and Conservation, University of Exeter, Cornwall Campus, Penryn, Cornwall TR10 9EZ, UK

**Keywords:** dispersal, life history, reproductive conflict, cooperation

## Abstract

Human females stop reproducing long before they die. Among other mammals, only pilot and killer whales exhibit a comparable period of post-reproductive life. The grandmother hypothesis suggests that kin selection can favour post-reproductive survival when older females help their relatives to reproduce. But although there is an evidence that grandmothers can provide such assistance, it is puzzling why menopause should have evolved only among the great apes and toothed whales. We have previously suggested (Cant & Johnstone 2008 *Proc. Natl Acad. Sci. USA* **105**, 5332–5336 (doi:10.1073/pnas.0711911105)) that relatedness asymmetries owing to female-biased dispersal in ancestral humans would have favoured younger females in reproductive competition with older females, predisposing our species to the evolution of menopause. But this argument appears inapplicable to menopausal cetaceans, which exhibit philopatry of both sexes combined with extra-group mating. Here, we derive general formulae for ‘kinship dynamics’, the age-related changes in local relatedness that occur in long-lived social organisms as a consequence of dispersal and mortality. We show that the very different social structures of great apes and menopausal whales both give rise to an increase in local relatedness with female age, favouring late-life helping. Our analysis can therefore help to explain why, of all long-lived, social mammals, it is specifically among the great apes and toothed whales that menopause and post-reproductive helping have evolved.

## 1. Introduction

In natural-fertility human populations, median ages at last birth cluster around 38 (Wood 1994; Cant & Johnstone 2008). This is followed by menopause some 10 years later. Yet, even in hunter–gatherer societies that do not enjoy the benefits of modern medicine, women can expect 20 or so years of active life following the cessation of reproduction (Hill & Hurtado 1996; Blurton-Jones *et al*. 2002). This pattern of early reproductive cessation and prolonged post-reproductive survival is very unusual. Chimpanzees and gorillas may, controversially, exhibit menopause (Nishida *et al*. 2003; Atsalis & Margulis 2006; Videan *et al*. 2006; Emery Thompson *et al*. 2007); more certainly, so too do short-finned pilot whales (*Globicephala macrorhynchus*) and killer whales (*Orcinus orca*; Whitehead & Mann 2000; McAuliffe & Whitehead 2005; Foote 2008). Short-finned pilot whale females stop breeding by 36 years of age, but can live up to 65 years (Kasuya & Marsh 1984; Marsh & Kasuya 1984); resident killer whale females stop breeding by 48 years old, but can live up to 90 years (Olesiuk *et al*. 1984; Foote 2008). By contrast, other long-lived mammals can continue to breed until the end of life—elephants, for instance, into their 60s (Moss 2001) and baleen whales into their 90s (Mizroch 1981).

The ‘grandmother’ hypothesis suggests that early reproductive cessation and post-reproductive survival have been favoured by kin selection. Older women, even if they do not bear more children themselves, can nevertheless gain inclusive fitness by helping their existing offspring to survive and reproduce (Williams 1957; Hamilton 1966; Hawkes *et al*. 1998). Similarly, it has been suggested that post-reproductive cetacean females may confer benefits on their social group, either directly (Baird 2000) or by virtue of the information they possess (Connor *et al*. 2000; Whitehead & Mann 2000; McAuliffe & Whitehead 2005). In humans, there is growing evidence that post-reproductive grandmothers can boost the fitness of their children (Sear *et al*. 2000; Voland & Beise 2002; Lahdenperä *et al*. 2004; Mace & Sear 2005; Fox *et al*. 2009), but the hypothesis itself raises two puzzling questions. First, among cooperatively breeding species in which there is an overlap of generations within the group, it is almost always younger individuals, who forego reproduction and assist older breeders (often their parents) as ‘helpers at the nest’ (Emlen 1995). What could account for the reversal of this typical pattern in menopausal species, in which older, post-reproductive ‘helpers’ assist younger breeders? Second, why should the reversed pattern have evolved only among the great apes and toothed whales? Why are post-reproductive grandmothers lacking in other long-lived, cooperative species?

We suggest that the answers to these questions may lie in the age-specific changes in local relatedness encountered by females in menopausal and non-menopausal species. Existing models of menopause often invoke selection to account for reproductive restraint (Williams 1957; Hamilton 1966; Rogers 1993; Hill & Hurtado 1996; Hawkes *et al*. 1998). To explain the timing of reproduction versus help, however, they focus exclusively on changes in the direct costs and benefits of reproduction with female age, while treating relatedness as a constant. But in species with overlapping generations, relatedness to other group members can also be expected to change with age as some group members disperse, and others die and are replaced. In a previous paper (Cant & Johnstone 2008), we showed that relatedness asymmetries owing to female-biased dispersal in ancestral humans are expected to favour younger females in reproductive competition with older females, providing an explanation for the timing of reproductive cessation. However, this argument seems to be inapplicable to cetacean menopause, since dispersal is not female-biased in menopausal whales. Here, however, we provide a much more general analysis of demographic effects on age-specific relatedness, which reveals that the social systems of menopausal cetaceans can also select for early reproductive cessation and late-life helping. By deriving general formulae for kinship dynamics, we highlight the underlying similarity between the ape and whale cases, which would otherwise be obscured by the differences in their social structure.

## 2. Results and discussion

To determine how relatedness and the impact of kin selection change across the lifespan, given different patterns of mating and dispersal, we build on the infinite-island modelling framework, which has been widely used to explore the consequences of demography for kin selection (Taylor 1992*a*,*b*; Van Balen & Rand 1998; Ronce *et al*. 2000; Taylor & Irwin 2000; Irwin & Taylor 2001; Lehmann & Perrin 2002; Gardner & West 2006; Lehmann & Keller 2007; Johnstone & Cant 2008). We focus on a diploid, sexually reproducing population that exists in a large number of discrete groups, each containing *n*_f_ female and *n*_m_ male breeders. Time proceeds in a series of discrete steps or cycles, during each of which the following events occur one after the other.

— *Reproduction*. Breeders mate and females produce a large number of offspring (we assume for simplicity a 1 : 1 sex ratio among offspring; qualitatively identical results are obtained when this assumption is relaxed (Johnstone & Cant 2008)). A fraction *m* of the young produced in a group are fathered by males within that group (chosen at random), the remainder by males from outside the group.
— *Dispersal*. Offspring disperse with probability *d*_f_ if female and *d*_m_ if male, each arriving in a random group if they do so.
— *Adult mortality*. Each breeder in a group survives with probability (1−*μ*_f_) if female and (1−*μ*_m_) if male.
— *Competition*. Offspring in a group, immigrant or native, compete on an equal basis with others of their own sex for any vacated breeding positions; those who fail to obtain a position, die.

The cycle then repeats. In appendix A in the electronic supplementary material, we derive the mean relatedness, to a female breeder of age *a*, of other females, and of males in her group. These relatedness coefficients will be denoted *r*_f_^*a*^ and *r*_m_^*a*^. Although several previous models have explored local relatedness and kin selection in a group-structured population with overlapping generations (Van Balen & Rand 1998; Taylor & Irwin 2000; Irwin & Taylor 2001) very few have explicitly considered the change in relatedness and kin selection with age; Ronce *et al*. (2000) do so but do not, however, explore the consequences of sex-biased dispersal as we do here.

**Figure 1.**
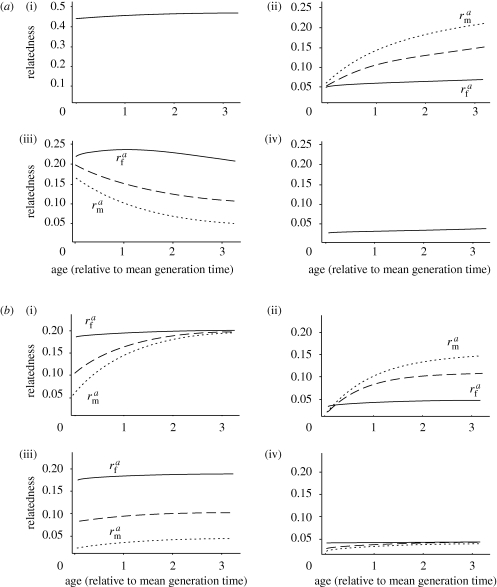
Age-specific local relatedness under different patterns of mating and dispersal. The mean relatedness to a breeding female of other females (solid lines), and of other males (dotted lines) in her group, is plotted as a function of her age. The dashed curves show mean relatedness to a female of other breeders, averaging across both sexes. We scale age relative to the mean generation time of the population (so that a value of one on the horizontal axis corresponds to *a* = 1/*μ*_f_). (*a*) Relatedness when mating occurs locally (*m* = 1), for all combinations of low (0.15) and high (0.85) female and male dispersal rates (*d*_f_ = *d*_m_ = 0.15; *d*_f_ = 0.15, *d*_m_ = 0.85; *d*_f_ = 0.85, *d*_m_ = 0.15; *d*_f_ = *d*_m_ = 0.85). (i) low female, low male dispersal; (ii) high female, low male dispersal; (iii) low female, high male dispersal; and (iv) high female, high male dispersal. (*b*) Relatedness when mating occurs outside the group (*m* = 0), again for all combinations of low and high female and male dispersal rates. (i) low female, low male dispersal; (ii) high female, low male dispersal; (iii) low female, high male dispersal; and (iv) high female, high male dispersal. In all cases, *μ*_f_ = *μ*_m_ = 0.1 and *n*_f_ = *n*_m_ = 3; we assume a small number of breeders because the group with which we will be concerned comprises only those directly affected by an individual female's reproductive decisions; in humans, for instance, we might focus on the ‘household’ or extended family unit, since post-reproductive grandmothers are of help chiefly to relatives (Sear *et al*. 2000; Voland & Beise 2002; Lahdenperä *et al*. 2004) and resources are preferentially shared within the family and with closer kin outside it (Kaplan & Hill 1985; Gurven 2004, 2006). The qualitative patterns shown, however, are unaffected by sex-differences in mortality or in the number of breeders per group.

As shown in figure 1, over the full parameter space of the model, relatedness among females shows little change with age. By contrast, relatedness between a female and local males may change with her age, sometimes dramatically, depending upon the pattern of mating and dispersal. Such age-specific changes occur when either there are sex-differences in dispersal, or when most mating is non-local; by contrast, if mating is local and both sexes exhibit similar dispersal rates, as typically assumed in existing models, there is no change with age (note that sex-differences in dispersal also lead to differences in relatedness between maternally and paternally inherited alleles (Haig 2000); here, we focus only on mean relatedness across both classes of allele).

When dispersal is male-biased, and mating occurs locally (figure 1*a*(iii)), then the relatedness between a female and local males decreases with her age. Under these circumstances, a female usually remains in her natal group with her father, so that initial relatedness to males is high. Any sons she or her local relatives produce, however, are likely to disperse away from the group. Consequently, as her father or other, older male relatives die, they are usually replaced by unrelated immigrants, and her relatedness to local males declines. Since female–female relatedness remains roughly constant over time, the result is that (averaging across both sexes) her relatedness to other local breeders, and to the offspring they produce, decreases with age.

By contrast, when males are philopatric, and either females disperse or mating is non-local or both (figure 1*a*(ii),*b*(i)(ii)), then the relatedness of males to a breeding female increases with her age. Under these circumstances, a female begins her reproductive life apart from her father (and paternal relatives), either because she has dispersed, or because she is the product of an extra-group mating. Initially, therefore, her relatedness to male breeders is low. Any sons she produces, however, are likely to remain in the group. Consequently, her relatedness to local males builds up over time. Since female–female relatedness remains roughly constant over time, the result is that (averaging across both sexes) her relatedness to other local breeders, and to the offspring they produce, increases with age.

What are the consequences of these age-dependent changes in relatedness for the evolution of reproductive behaviour? Recent models have shown that the impact of kin selection on social acts depends on the level of competition between relatives, which is determined by population structure and patterns of dispersal (Taylor 1992*a*; Ronce *et al*. 2000; Gardner & West 2006; Lehmann & Keller 2007; Johnstone & Cant 2008). Consider, then, an action taken by a female of age *a* that entails an immediate loss of *c* offspring for the actor, and an immediate gain of *b* offspring for others in the group (note that the benefit of the act is conferred on all members of the local group, rather than being targeted specifically at a female's relatives). If *b* > 0, we will speak of ‘helping’ behaviour, while if *b* < 0, we will speak of ‘harming’ behaviour. These immediate losses and gains will further impact on the fitness of locally produced offspring (both those of the actor and of others) through competition for breeding vacancies. Using an inclusive fitness approach (Taylor 1992*a*,*b*) in the electronic supplementary material, appendix B, we quantify the impact of the act in question on the actor and on local females and males for three illustrative cases: (i) male-biased dispersal and local mating, (ii) female-biased dispersal and local mating, and (iii) no dispersal of either sex but non-local mating. Together with the age-specific relatedness coefficients derived in the electronic supplementary material, appendix A, this allows us to determine the strength of selection favouring helping and harming at different ages.

We focus on helping and harming behaviour that affect all others in the local group, because we are concerned with the evolution of menopause and reproductive cessation. Reproduction is likely to impose fecundity costs on all other local breeders, because it intensifies local competition among breeders for food and other resources to support offspring production (in addition to intensifying competition for local breeding vacancies among offspring that remain on the patch). Consequently, it constitutes perhaps the simplest form of harming behaviour. Conversely, if reproductive cessation reduces the intensity of resource competition experienced by other local breeders, leading to increase in their fecundity (in addition to reducing competition for local breeding vacancies among offspring that remain on the patch), it constitutes a simple form of indiscriminate helping behaviour.

Using the above approach, we find that in all cases, the act in question will be favoured only if the magnitude of the ratio *c/b* is sufficiently small, i.e. only if the immediate cost incurred by the actor is sufficiently small compared with the immediate impact of the act (whether positive or negative) on the recipients. Whether it is helping or harming that is potentially favoured, however, and how low must the magnitude of *c/b* be for this to occur, changes with age in a manner that depends upon the pattern of mating and dispersal.

**Figure 2.**
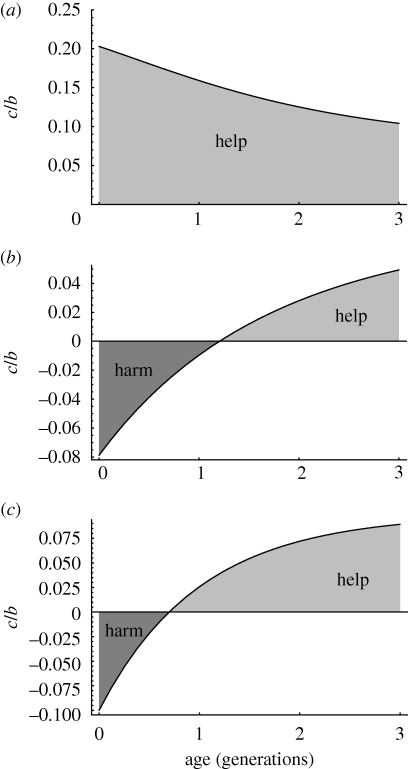
Age-specific kin selection under different patterns of mating and dispersal. The graphs show the absolute magnitude of the ratio *c/b* below which a social action may be favoured in females of different ages, in three illustrative cases: (*a*), dispersal is male-biased (*d*_f_ = 0.15, *d*_m_ = 0.85) and mating is local (*m* = 1), yielding a decrease in local relatedness with female age; (*b*), dispersal is female-biased (*d*_f_ = 0.85, *d*_m_ = 0.15) and mating is local (*m* = 1), yielding an increase in local relatedness with female age; and (*c*), neither sex disperses (*d*_f_ = *d*_m_ = 0) but mating is non-local (*m* = 0), again yielding an increase in local relatedness with female age. Positive values indicate that selection will favour helping behaviour when *c/b* falls above zero but below the value shown (in the lightly shaded area), while negative values indicate that selection will favour harming behaviour when *c*/*b* falls below zero but above the value shown (in the heavily shaded area). Where mating occurs locally, helping and harming behaviours affect the number of offspring produced jointly by local males and females. Where mating occurs outside the group, however, helping and harming behaviour might impact on the number of offspring produced within the group by local females, or on the number of offspring sired outside the group by local males. For simplicity, we have assumed in this case that help or harm is evenly distributed between male and female recipients. In fact, the model predicts that selection will favour helping of males rather than females, because production of additional offspring within the group intensifies local competition for breeding vacancies. Whether or not help is directed preferentially at males, however, does not change the fact that selection favours harming less strongly and/or helping more strongly in older individuals.

In figure 2, we see that when dispersal is male-biased and mating occurs locally (panel *a*), yielding a decrease in local relatedness with female age (as described above), selection favours helping more weakly for older than for younger females (i.e. the critical ratio of cost to benefit below which an altruistic act is favoured declines with age). The reason is that older females are less related on average to the offspring they help to produce than are younger females. Since breeding entails relatively greater kin-selected costs, and helping yields relatively greater kin-selected benefits at younger ages, helping (if it evolves) is more likely to be restricted to younger individuals and breeding to older ones (note that helpers must have someone to help). By contrast, when males are philopatric and either females disperse or mating is non-local (panels *b* and *c*), yielding an increase in local relatedness with female age (as described above), we see that helping behaviour is favoured more strongly at older ages, because older females are more closely related to the offspring they help to produce than are younger females. Indeed, below a critical age selection may in fact favour harming rather than helping behaviour, as a means of reducing local competition for breeding spots. Since breeding entails relatively greater kin-selected costs, and helping yields relatively greater kin-selected benefits at older ages, helping is in this case more likely to be restricted to older individuals and breeding to younger ones. In short, whether it is younger or older individuals that are most likely to refrain from breeding and adopt the role of kin-selected helpers, depends upon the pattern of dispersal and mating.

In addition to the above, where mating occurs outside the group (and one or both sexes exhibit a degree of philopatry), selection will favour helping of males rather than females, because production of additional offspring within the group intensifies local competition for breeding vacancies (note that we have focused on the impact of kin selection on female behaviour; patterns of age-specific local relatedness experienced by males are, in the case of local mating, the precise mirror image of those experienced by females, but males will not experience the same age-specific change in the impact of kin selection because competition over paternity does not directly impact on female fecundity, and it is relatedness to local members of the opposite sex that changes with age).

We previously (Cant & Johnstone 2008) showed that the outcome of reproductive competition between generations will depend strongly on whether older females compete with their daughters or with their sons' unrelated mates. Our analysis relied, however, on extrinsically specified patterns of relatedness, and implicitly assumed that there were only two age-classes of female as well as only two competing breeders per patch. In addition, we did not consider the possibility of non-local mating. The present analysis shows that we can derive age-specific coefficients of relatedness from patterns of dispersal and mating very generally, and thus predict which demographies are more or less favourable to the evolution of late-life helping.

Most social mammals exhibit male-biased dispersal and female-biased philopatry (Clutton-Brock 1989; Lawson Handley & Perrin 2007). By contrast, three lines of evidence suggest an evolutionary history of female-biased dispersal and male-biased philopatry in humans. First, transfer between groups is markedly female-biased in our closest relatives: chimpanzees (both *Pan troglodytes* (Pusey *et al*. 1997; Nishida *et al*. 2003; Langergraber *et al*. 2007) and *Pan paniscus* (Eriksson *et al*. 2006), and gorillas (Yamagiwa & Kahekwa 2001; Stokes *et al*. 2003) exhibit strongly female-biased dispersal. Second, molecular analysis of Y-chromosome and mitochondrial DNA (mtDNA) variation suggests a history of female-biased transfer in most human populations (Sielstad *et al*. 1998; Hammer *et al*. 2001; Segurel *et al*. 2008), at least at the relevant, local scale (Wilder *et al*. 2004). Comparison of mtDNA and nuclear DNA variation in humans, chimpanzees and gorillas supports a shift to female-biased dispersal in the *Pan-Homo* lineage, after divergence from gorillas (Jensen-Seaman *et al.* 2001). Finally, among human forager societies female transfer to the husband's family at marriage or after the early years of marriage is more common than the reverse pattern (Ember 1978; Marlowe 2004), although flexible residence and bilocality are also common (Alvarez 2004). Although far from conclusive, these different lines of evidence suggest that menopause is likely to have evolved in a social environment in which dispersal was female-biased (Cant & Johnstone 2008; Cant *et al*. 2009). In contemporary and historical human populations, grandmothers often assist the offspring of both sons and daughters (Sear & Mace (2008) report that 60% of statistically valid studies found a positive impact of paternal grandmothers on offspring survival, and 69%, a positive impact of maternal grandmothers). However, since the impact of different kin classes varies among populations, patterns of helping do not offer a reliable guide to ancestral patterns of dispersal.

Turning to the menopausal cetaceans, both male and female resident killer whales are philopatric, but mate outside the local group (Baird 2000; Whitehead & Mann 2000; it is not known whether or not transient killer whales, which do disperse, also exhibit menopause). Short-finned pilot whales are thought to show a similar pattern—there is clear evidence that this is the case for their sister species, the long-finned pilot whale (Amos 1993), and the available genetic data suggest that the short-finned and the long-finned species are comparable (Amos 1998).

Our analysis thus implies that females of most social mammalian species will experience a decline in local relatedness with age, but that the two unusual and very different social arrangements that characterize menopausal species (respectively, female-biased dispersal and local mating in ancestral humans, and philopatry of both sexes combined with extra-group mating in pilot and resident killer whales) both give rise to an increase in local relatedness with female age. This build-up of local relatedness over the reproductive lifespan of a female means that the great apes and toothed whales, by contrast with most mammals, are predisposed to the evolution of reproductive restraint and altruistic helping behaviour later rather than earlier in life. The value of an explicit focus on kinship dynamics is that it reveals the underlying similarity between the ape and whale cases, which would otherwise be obscured by the differences in their social structure.

Under the ‘whale’ model (Figure 2*c*), late-life helping is driven by increasing local relatedness to group males as a female grows older. Moreover, because mothers experience local resource competition when females in the group reproduce, while local males' offspring are raised in other groups, the model predicts that females (and especially older females) should preferentially direct care towards males rather than towards females. Two observations offer empirical support for this prediction. First, in resident killer whales, mothers maintain closer associations with their adult sons than with their adult daughters, and may aid their sons' foraging efforts, or form effective alliance partners for them in agonistic encounters with other males (Baird 2000). Second, whaling reports suggest that in pilot whales daughters are weaned at around 4–6 years old, whereas sons continue to suckle into their teens (Kasuya & Marsh 1984; Mann 2000).

In addition to discriminating between sons and daughters, females in both the ape and whale cases would be expected to selectively help closer relatives within the group. In our analysis, however, we have assumed that helping and harming affect all other members of the group equally. As we point out, refraining from reproduction is likely to confer at least some indiscriminate benefits on all other members of the local group, owing to reduced competition among breeders for resources. Nevertheless, it would be interesting to model discriminatory helping or harming, though to do so is technically challenging and beyond the scope of this paper. We emphasize, however, that the kinship dynamics we have explored are likely to influence selective helping and harming of close relatives in a similar way to the generalized helping and harming that we model here. Changes in mean relatedness with age largely reflect changes in the expected number of close relatives present in the group. Consequently, even though a female's relatedness to her daughters and grandchildren, or to her parents and grandparents, remains constant throughout her lifespan, the expected numbers of close relatives present in the local group, and thus the opportunity to help close relatives, will change with age in a way that corresponds closely to changes in mean relatedness. The expected benefits of ceasing reproduction in order to selectively assist close kin will thus increase with age in the ape and whale cases because younger females are less likely than are older females to have close relatives in the group—there is little value to sacrificing the possibility of direct reproduction to become a helper at an age when there are likely to be few or no kin present to help.

To conclude, our analysis shows that age-specific changes in relatedness can have a significant influence on the evolution of reproductive life history, and reveals the underlying similarity between the ape and whale cases in this respect, which would otherwise be obscured by the differences in their social structure. We emphasize that a species' pattern of mating and dispersal is not the only factor relevant to the evolution of ‘grandmothering’ and menopause. Not only age-specific relatedness but also the inclusive fitness costs and benefits of breeding and helping will vary from one species to another. For instance, the costs of reproductive competition are likely to be greater in humans than in chimpanzees and gorillas because of the greater cost of offspring production and more extensive sharing of resources (Cant & Johnstone 2008). Consequently, we would not predict that every species with male-biased philopatry will inevitably evolve menopause. Nevertheless, kinship dynamics can help to explain why, of all long-lived, social mammals, it is specifically among the great apes and toothed whales that menopause has evolved.
